# Evolution of Traumatic Parenchymal Intracranial Hematomas (ICHs): Comparison of Hematoma and Edema Components

**DOI:** 10.3389/fneur.2018.00527

**Published:** 2018-07-04

**Authors:** Sean Wilkes, Erin McCormack, Kimbra Kenney, Brian Stephens, Ross Passo, Leah Harburg, Erika Silverman, Carol Moore, Tanya Bogoslovsky, Dzung Pham, Ramon Diaz-Arrastia

**Affiliations:** ^1^Department of Behavioral Health, Tripler Army Medical Center, Honolulu, HI, United States; ^2^Center for Neuroscience and Regenerative Medicine, Uniformed Services University of the Health Sciences, Bethesda, MD, United States; ^3^Department of Neurology, Penn Presbyterian Medical Center, University of Pennsylvania, Philadelphia, PA, United States

**Keywords:** TBI, edema, hematoma, CT, neurotrauma

## Abstract

This study seeks to quantitatively assess evolution of traumatic ICHs over the first 24 h and investigate its relationship with functional outcome. Early expansion of traumatic intracranial hematoma (ICH) is common, but previous studies have focused on the high density (blood) component. Hemostatic therapies may increase the risk of peri-hematoma infarction and associated increased cytotoxic edema. Assessing the magnitude and evolution of ICH and edema represented by high and low density components on computerized tomography (CT) may be informative for designing therapies targeted at traumatic ICH. CT scans from participants in the COBRIT (Citicoline Brain Injury Trial) study were analyzed using MIPAV software. CT scans from patients with non-surgical intraparenchymal ICHs at presentation and approximately 24 h later (±12 h) were selected. Regions of high density and low density were quantitatively measured. The relationship between volumes of high and low density were compared to several outcome measures, including Glasgow Outcome Score—Extended (GOSE) and Disability Rating Score (DRS). Paired scans from 84 patients were analyzed. The median time between the first and second scan was 22.79 h (25%ile 20.11 h; 75%ile 27.49 h). Over this time frame, hematoma and edema volumes increased >50% in 34 (40%) and 46 (55%) respectively. The correlation between the two components was low (*r* = 0.39, *p* = 0.002). There was a weak correlation between change in edema volume and GOSE at 6 months (*r* = 0.268, *p* = 0.037), change in edema volume and DRS at 3 and 6 months (*r* = −0.248, *p* = 0.037 and *r* = 0.358, *p* = 0.005, respectively), change in edema volume and COWA at 6 months (*r* = 0.272, *p* = 0.049), and between final edema volume and COWA at 6 months (*r* = 0.302, *p* = 0.028). To conclude, both high density and low density components of traumatic ICHs expand significantly in the first 2 days after TBI. In our study, there does not appear to be a relationship between hematoma volume or hematoma expansion and functional outcome, while there is a weak relationship between edema expansion and functional outcome.

## Introduction

Traumatic brain injury (TBI) is a major worldwide health concern, affecting approximately 1.7 million individuals in the United States annually (1), and costing an estimated $76.5 million (2). Additionally, TBI is a major risk for military personnel currently deployed to combat theaters, with approximately 4.9% of service members demonstrating symptoms upon redeployment (3).

Intracranial hematoma (ICH) is a common consequence of TBI. It may be extra-axial, as in the case of epidural or subdural hematomas, or intra-axial as in the case of intraventricular and intraparenchymal hematomas. Intracranial hematomas can be a medical emergency as they can cause raised intracranial pressure, resulting in physical damage to brain tissue, while blood loss and interruption of flow may cause focal ischemia, sufficient to independently cause cell damage and death.

In addition to the high-density hematoma component, intraparenchymal hemorrhages are frequently accompanied by a surrounding low-density edema component. In the context of TBI, edema is thought to arise as a consequence of a complex series of molecular and cellular events, involving neuronal, vascular, glial, and support cells within the brain (4). The etiology of TBI-related edema may be divided into three categories: (1) Vasogenic edema- caused by injury to capillary endothelial tissue, increasing cellular permeability and disruption of the blood brain barrier (BBB). Protein-rich fluid from the blood diffuses into the interstitial space, increasing its volume; (2) Osmotic edema- caused by an alteration of and imbalance in the osmotic gradient between the plasma and surrounding tissues, as may be seen in conditions of serum hypoosmolarity or when severe ischemic necrosis resulting in localized hyperosmolarity of cerebral tissue; (3) Cytotoxic edema- caused by any combination of three following functional/metabolic changes in astrocytes and neurons as a result of injury: the increased permeability of the cellular membrane to sodium and potassium, the failure of active ion transporters due to ATP depletion, or the sustained uptake of neurotransmitters and metabolites.

Traditionally, edema following TBI was thought to be primarily vasogenic in nature. However, this view has been challenged by studies that indicate only limited alterations to and disruption of the BBB following TBI (5–10) and that a disrupted BBB alone is neither sufficient nor necessary for edema formation to occur (11) suggesting that initial edema formation after TBI is primarily cytotoxic in nature. Furthermore, MRI studies in TBI with focal edema demonstrated a reduction in the apparent diffusion coefficient in the associated regions of injury, which is highly suggestive of cytotoxic edema (10, 12). Other studies have suggested that vasogenic edema may predominate early post-injury, and cytotoxic edema predominates later in the hours to days following TBI (13–16).

Early expansion of ICH occurs in approximately 50% of TBI cases (17), but most previous studies have measured the high density (blood) component only (18–21). ICHs are usually associated with a perihematomal low density component, which at least in part represents cytotoxic edema, and one recent study has demonstrated expansion of this perihematomal component in animal models (22). Hemostatic therapies aimed at preventing re-bleeding may increase the risk of perihematoma thrombosis and infarction, thereby increasing cytotoxic edema. Alternatively, the design of clinical trials of drugs targeting cytotoxic edema requires detailed knowledge about the contribution of cytotoxic edema to functional outcome after TBI. Assessing the relative evolution of both high and low density components may be informative for designing medical therapies aimed at improving outcome after traumatic ICH.

The long-term effects of edema formation in TBI or its relationship to known sequelae of TBI remain uncertain. As noted, cytotoxic edema is a prominent component of edema formation and is thought to result from energy failure, inability to maintain ionic gradients across neural cell membranes, and subsequent cell death. Thus, edema may represent cytotoxicity and reflect functional deficits during and after recovery.

This study was designed to test three hypotheses: (1) Both the high density and low density component of traumatic ICHs expand over the first day after injury; (2) There is significant correlation between expansion of the two ICH components; and (3) Initial volume and expansion of the edema component of traumatic ICHs are associated with functional outcome.

## Materials and methods

We quantitatively assessed the evolution of traumatic ICH over the first approximately 24 h after injury. CT scans from participants in the COBRIT study (23) were selected based on several inclusion and exclusion criteria. For inclusion, CT imaging had to be available for subject at time of presentation and 12–36 h after presentation, and intraparenchymal hemorrhage of >1 cm in diameter (corresponding to a volume of 80 mm^3^) had to be present. Subjects were excluded if the CT images were of poor quality. Since we were interested in the effect of parenchymal ICHs, patients were excluded if the predominant pathology was extra-axial, defined as a volume of extra-axial ICH more than twice the parenchymal ICH volume. Since our focus was on elucidating information to inform clinical trials of medical therapies for traumatic ICHs, we excluded patients who required a neurosurgical procedure immediately after the initial scan.

Of the 1,213 COBRIT study subjects available for this analysis, 312 were identified with intraparenchymal lesions. Fifty-five patients were excluded because they required a craniectomy after the first scan, and an additional 99 were excluded because they lacked the necessary imaging data or their stored images were unusable for this study. Of the 158 subjects with usable imaging data at presentation and again between 12 and 36 h later, another 74 were excluded because the ICH was too small or extra-axial hematomas were the predominant feature, leaving 84 subjects for evaluation. The median time between the first and second scan was 22.79 h (25%ile 20.11 h; 75%ile 27.49 h).

**Figure 1:**
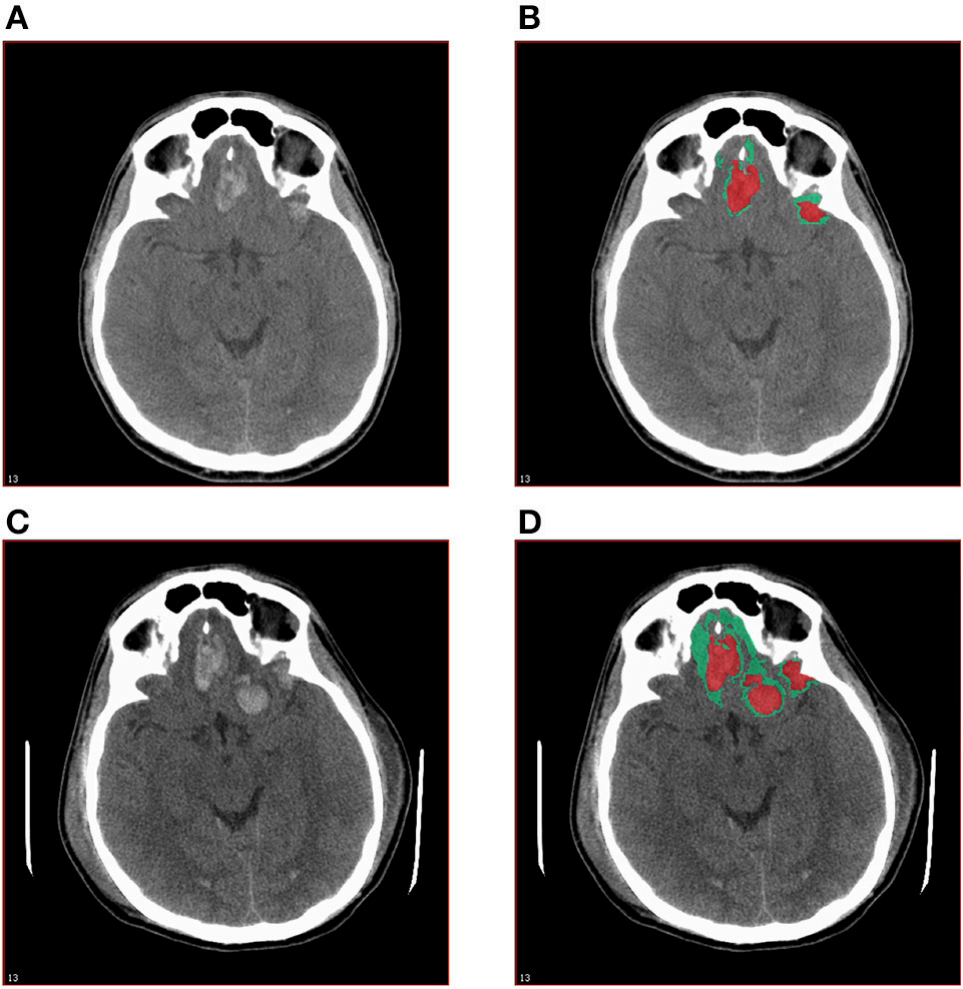
Quantitative measurement of high density and low density components of traumatic ICH. The base layer **(A)** and annotated **(B)** images of the initial CT scan and the base layer **(C)** and annotated **(D)** images of the CT scan at 12–36 h post-injury are shown.

Scans were blinded with respect to time of scan and randomly assigned to one of three raters, with 10% overlap to ensure reliability. The CT scans were analyzed using MIPAV software. Regions of high density, (corresponding to blood) and low density (corresponding to perihematomal edema) were identified through visual inspection assisted by measurements of quantified radiodensity, then manually delineated and their volumes measured (Figure 1). Measurements were confined to the parenchyma and extra-axial densities suggestive of minor epidural or subdural hematoma or edema were ignored. Edema and hematoma size and rate of expansion were then compared against several outcome measures, as follows: Glasgow Outcome Score—Extended (GOSE), Disability Rating Score (DRS), Brief Symptom Inventory with Global Severity Index (GSI), Positive Symptom Distress Index (PSDI), Controlled Oral Word Association (COWA), and Satisfaction with Life Scale (SWLS). Associations were statistically evaluated using Spearman's correlation.

## Results

**Figure 2:**
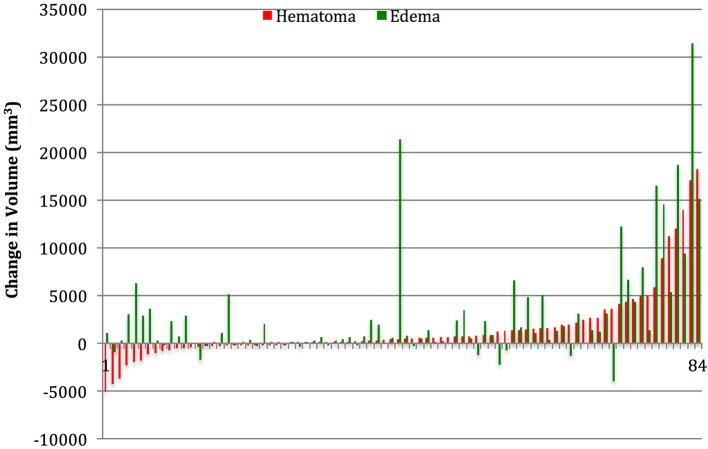
Subject by subject relationship between change in hematoma and change in edema volume. In the first 12–36 h after presentation, hematoma, and edema volumes increased >50% in 34 (40%) and 46 (55%) respectively.

Table 1 summarizes the demographic data for patients included in the study. They were predominantly males in their late 30's, and on average had a moderate TBI based on admission Glasgow Coma Scale (GCS). Table 2 summarizes the measurements of hematoma and edema component noted on the initial CT, the CT performed approximately 24 h later (±12 h), and the change in volumes between the two time points. The mean change in hematoma volume was 1,556 mm^3^ with a median of 472 mm^3^, while the mean change in edema volume was 2,844 mm^3^ with a median of 764 mm^3^ (Table 2). Hematoma volume increased more than 50% in 34 subjects (40%), and edema volume increased more than 50% in 46 subjects (55%). The correlation between expansion of the hematoma component and expansion of the edema component was low (*r* = 0.39, *p* = 0.002) (Figure 2). Six-month GOSE scores were available for 61 subjects. There were no significant correlations between hematoma or edema volumes at baseline or at 24 h (±12 h) and GOSE at 6 months (Table 3). There was a weak correlation between change in edema volume and GOSE at 6 months (*r* = 0.268, *p* = 0.037).

**Table 1:** Demographics of subjects included in the study.

**Gender**	
Male	77%
Female	23%
**Race**
White	90.4%
African American	5.9%
Asian	1.2%
Hawaiian/Pacific Islander	1.2%
Mixed Race	1.2%
**Age**
Median	35
Max	69
Min	18
Average	38.1
SD	15.0
**Admission GCS**
Median	9
Max	15
Min	3
Average	9.1
SD	5.3
**GOSE at 180 days**
Median	6
Max	8
Min	1
Average	6
SD	1.6

*GCS, Glasgow Coma Scale; GOSE, Glasgow Outcome Scale Extended*.

**Table 2:** Descriptive statistics of hematoma and edema volumes.

**ICH component**	**Volume at 0 h (mm^3^)**	**Vol. at 12–36 h (mm^3^)**	**Change in volume (mm^3^)**
**HEMATOMA**			
Mean	3,634	5,190	1,556
SEM	605	821	427
Median	1,676	2,519	472
25%tile	622	880	−154
75%tile	4,658	5,957	1,602
**EDEMA**			
Mean	2,566	5,410	2,844
SEM	512	815	605
Median	1,142	2,305	764
25%tile	295	777	25
75%tile	2,357	6,272	3,086

**Table 3:** Spearman's rho correlation of edema and hematoma measurements with outcomes.

**Spearman's rho comparison (*N*)**		**Edema Δ volume**
Hematoma Δ volume (84)	Coefficient (ρ)	**0.390**
	*p*-value (α)	0.002
GOSE 30 days (70)	Coefficient (ρ)	0.154
	*p*-value (α)	0.203
GOSE 90 days (72)	Coefficient (ρ)	0.176
	*p*-value (α)	0.139
GOSE 180 days (61)	Coefficient (ρ)	**0.268**
	*p*-value (α)	0.037
DRS 90 days (71)	Coefficient (ρ)	**-0.248**
	*p*-value (α)	0.037
DRS 180 days (59)	Coefficient (ρ)	**-0.358**
	*p*-value (α)	0.005
GSI 90 days (64)	Coefficient (ρ)	−0.036
	*p*-value (α)	0.777
GSI 180 days (53)	Coefficient (ρ)	−0.054
	*p*-value (α)	0.700
PSDI 90 days (64)	Coefficient (ρ)	−0.016
	*p*-value (α)	0.900
PSDI 180 days (53)	Coefficient (ρ)	−0.104
	*p*-value (α)	0.459
COWA 90 days (65)	Coefficient (ρ)	0.234
	*p*-value (α)	0.061
COWA 180 days (53)	Coefficient (ρ)	**0.272**
	*p*-value (α)	0.049
SWLS 90 days (62)	Coefficient (ρ)	0.171
	*p*-value (α)	0.184
SWLS 180 days (53)	Coefficient (ρ)	0.024
	*p*-value (α)	0.865

*The correlation between expansion of the hematoma component and expansion of the edema component was low (r = 0.390, p = 0.002). There were weak correlations between change in edema volume and GOSE at 6 months (r = 0.268, p = 0.037), between change in edema volume and DRS at 3 months (r = −0.0248, p = 0.037) and at 6 months (r = 0.358, p = 0.005), between final edema volume and COWA at 6 months (r = 0.302, p = 0.028), and between change in edema volume and COWA at 6 months (r = 0.272, p = 0.049). Only the correlation between the expansion of the hematoma and edema (p = 0.002) and the correlation between change in edema volume and DRS (p = 0.005) survived Bonferroni correction for multiple comparisons. GOSE, Glasgow Outcome Score—Extended; DRS, Disability Rating Score; GSI, Global Severity Index; PSDI, Positive Symptom Distress Index; COWA, Control Oral Word Association; SWLS, Satisfaction with Life Scale. The bolded values represent statistically significant correlations*.

Six-month DRS scores were available for 59 subjects. There were no correlations between hematoma or edema volumes at baseline or 24 h later (±12 h) and DRS at 6 months. There were a weak correlations between change in edema volume and DRS at 6 months (*r* = 0.358, *p* = 0.005).

Six-month COWA scores were available for 59 subjects. There were no correlations between initial or final hematoma volumes and COWA at 6 months. There was a weak correlation between final edema volume and COWA at 6 months (*r* = 0.302, *p* = 0.028). There was also a weak correlation between change in edema volume and COWA at 6 months (*r* = 0.272, *p* = 0.049).

No statistically significant associations were found between edema or hematoma volumes or change in volumes and patient age, Glasgow Coma Scale score (GCSs), or Injury Severity Scale score (ISSs).

Only the correlation between the expansion of the hematoma and edema (*p* = 0.002) and the correlation between change in edema volume and DRS (*p* = 0.005) survived Bonferroni correction for multiple comparisons.

## Discussion

Both the high density (blood) and low density (edema) components of traumatic ICHs expand significantly in the first 24 h after TBI, and the portion of patients experiencing significant expansion of edema component is similar to the portion experiencing significant expansion of the hematoma component. This is consistent with previous studies, which suggest that the presence of blood may contribute to edema formation, and that the volume of perihematomal edema correlates with the volume of lysed blood cells present (24, 25). However the correlation between the expansion of edema and hematoma in our study was low (Figure 2), supporting the notion that other processes equally contribute to edema formation, such as ischemic injury or other metabolic insults (26). This is a novel finding, and indicates that hemostatic control may be necessary but not sufficient to prevent edema formation and resultant pathophysiological processes, such as rise in intracranial pressure.

With respect to outcomes, previous studies have demonstrated an association between the presence of hematoma expansion or progression and increased morbidity (17, 27–29) however they did not quantify the degree of expansion nor account for the presence and expansion of perihematomal edema.

Our research found no relationship between hematoma volume or expansion and functional outcome at 6 months after injury. This may be due to the relatively small size of traumatic ICHs analyzed in our study. We chose to exclude patients with large ICHs that required surgical therapy, as our goal was to investigate measures that would be useful for the design of clinical trials of medical therapies for traumatic ICH.

We did demonstrate associations between edema expansion and three measures of functional outcome: GOSE, COWA, and DRS at, 6 months after injury. These correlations indicate a weak relationship. However, given the limitations of the study and considering the comparative absence of any association between hematoma volume and outcome, these results suggest that edema expansion may prove superior to other measures as a prognostic indicator for traumatic ICH.

The fact that associations were observed with certain outcome measures but not others bears some consideration. The correlations found between edema expansion and GOSE and DRS, while statistically significant, were weak to begin with. Both GOSE and DRS assess measurable neurological symptoms - such as eye opening, communication/speech, and motor symptoms—in conjunction with measures of basic functionality such as feeding, grooming, toileting, and dependency of others. These functional assessments are likely able to capture a broad range of neuropsychiatric deficits with the expectation that they will lead to a narrower range of common, overlapping functional deficits, following TBI.

The GSI and the Positive Symptom Distress Index, by contrast, are both derived from the Brief Symptom Inventory, which is a series of 53 items covering a broad domain of largely psychiatric symptoms. Thus, they may not be sufficiently generalizable as sequelae of traumatic brain injury, or the symptoms they measure may not be sufficiently common enough in the months immediately following traumatic brain injury, for an association to be found.

This study exhibited some significant limitations. First, our primary interest in this research was in evaluating traumatic ICHs that did not require surgical management. As discussed above, we excluded all ICHs requiring surgical intervention because edema and hematoma expansion could no longer be followed longitudinally and our intent was to investigate imaging measures that would inform future clinical trials of medical therapies for ICH. Non-surgical ICHs tend to be smaller and have better outcomes. ICHs requiring surgical interventions may demonstrate more significant associations between hemorrhage and outcomes but were not included in our analysis. Another limitation is that most ICHs were relatively small, and the number of moderate-sized ICHs (between 5,000 and 20,000 mm^3^) was too small to further analyze. The small size of most of the ICHs in our study may limit the generalizability of our conclusions to moderate to large ICHs which do not require immediate neurosurgical intervention. Additionally the small sizes of these ICHs make their measurement prone to variability due to variability in the distance between CT cuts among different scans. This may account for the reduction in hematoma and edema size observed in some patients over the first approximately 24 h.

Second, the quality and resolution of CT scans are such that while hematomas are fairly easy to visually delineate given that acute blood typically presents in the range of 50–100 hounsfields units (HUs), which contrasts well against brain tissue, the visual delineation of edema is comparatively more difficult, as areas of edema are often only 2–6 HUs lower than surrounding non-edematous tissue. Thus the measurements of edema volumes may be less precise than the measurement of hematoma volumes. In future studies we hope to evaluate the reliability of the measurement of edema on CT via comparison to measures performed with MRI.

Third, the variability in the time at which the second CT scan was taken, which occurred between 12 and 36 h after the initial CT scan, presents another major limitation since the degree of expansion is heavily influenced by the time since initial injury. If the rate of expansion between hematoma and edema differs, the differing time points at which the ICHs were measured may explain the poor observed relationship between hematoma and edema rates of expansion.

Another limitation is the lack of trend data regarding blood pressure, hematocrit, and coagulation parameters. Collection of such data in future prospective studies will help ascertain the contribution of these factors to evolution of parenchymal contusions, and will provide guidance for management. Another major limitation was the variability in the location of injury within the brain parenchyma. Some injuries were isolated to the frontal lobe, others temporal or parietal. Some affected peripheral gray matter or cortical white matter tracts while others affected more central structures. Still others were multi-lobar or spread diffusely about the parenchyma. Given that these locations represent distinct functional areas, outcome measures are undoubtedly influenced by the site of injury, which introduces a potentially significant confounder. Larger subject numbers in future studies may allow for considerations based on the region of brain injury and an analysis of hematoma and edema expansion localized to a specific region.

## Ethics statement

This study was carried out in accordance with the recommendations of FWA 00001628 and DoD Assurance P60001, and was declared an EXEMPT protocol by the Uniformed Services University Institutional Review Board. A de-identified data set and de-identified CT scans were utilized for this study, and at no time did the investigators have access to identifiable information or to any linking codes that could identify participants. The protocol was approved by the Uniformed Services University Institutional Review Board.

## Author contributions

SW: co-investigator, lead author, primary researcher and study coordinator. EM, RP, LH, and ES: extracted, prepared, and analyzed neuroimaging data. KK: provided faculty oversight and guidance of research, instructed in the analysis of neuroimaging data. BS: performed statistical analysis. CM: provided technical and administrative support for the coordination of study. TB: provided faculty oversight and guidance of research, instructed in the analysis of neuroimaging data. DP: provided faculty oversight and guidance of research, instructed in the analysis of neuroimaging data. RD-A: principal investigator.

### Conflict of interest statement

The authors declare that the research was conducted in the absence of any commercial or financial relationships that could be construed as a potential conflict of interest.
